# Five year outcomes of Boston type I keratoprosthesis as primary versus secondary penetrating corneal procedure in a matched case control study

**DOI:** 10.1371/journal.pone.0192381

**Published:** 2018-02-06

**Authors:** Kai B. Kang, Faris I. Karas, Ruju Rai, Joelle A. Hallak, Joann J. Kang, Jose de la Cruz, Maria S. Cortina

**Affiliations:** Illinois Eye and Ear Infirmary, Department of Ophthalmology and Visual Sciences, University of Illinois at Chicago, Chicago, IL, United States of America; Massachusetts Eye & Ear Infirmary, Harvard Medical School, UNITED STATES

## Abstract

Despite improved retention and reduced complication rates paving the way for the current expansion of applications and surge in prevalence for the Boston type I Keratoprosthesis (KPro), the most frequent indication for its implantation today remains prior graft failure. The purpose of this study is to evaluate the long-term outcomes of primary KPro and compare to secondary implantation in a matched cohort study. This study included patients who underwent KPro implantation in a single center by two surgeons between July 2008 and October 2014. All eyes with KPro implantation as the primary procedure with a minimum follow up of 12 months were matched with eyes with same preoperative diagnoses that underwent secondary KPro implantation. Main outcomes included visual acuity and device retention. A total of 56 eyes were included with 28 eyes in each group. Mean follow up was 5.0 years for both groups. Twenty-nine percent (8) of the eyes in the primary group had a diagnosis of chemical or thermal injuries, 25% (7) aniridia, 18% (5) autoimmune disease, 4% (1) infectious keratitis/neurotrophic cornea, 7% (2) gelatinous corneal dystrophy, 7% (2) ectrodactyly ectodermal dysplasia/limbal stem cell deficiency, and 11% (3) uveitis/hypotony. Sixty-one percent (17) of the eyes in the primary group and 39% (11) in the secondary group maintained a final best-corrected visual acuity of 20/200 or better at a mean follow up of 5.0 years; the probability of maintaining best-corrected vision is 0.83 and 0.49 for primary and secondary groups at 5.0 years (p = 0.02). There is no statistically significant difference between groups in device retention (p = 0.22) or postoperative complication rates (p >0.05). This study demonstrates that Boston KPro implantation may be successful as a primary procedure in patients at high risk of failure with traditional penetrating keratoplasty. The device has a good long-term retention rate and visual outcomes are promising however a larger study is needed for more definitive results.

## Introduction

First approved by the U.S. Food and Drug Administration in 1992, the Boston type 1 Keratoprosthesis (KPro) has undergone many iterative modifications in device design, surgical technique, postoperative management and reduced rates of complications [1–3]. This artificial cornea has now become a legitimate, well-established tool in our armamentarium for treating patients with debilitating corneal blindness[4–7]. KPro surgery has traditionally been thought of as a procedure of last resort. Despite improved retention and reduced complication rates paving the way for the current dramatic expansion of applications and surge in prevalence for the KPro, the most frequent indication for its placement today remains prior graft failure[4,5,8–12].

We posit that there is scope for the more preemptive use of the KPro as the therapy of choice (primary KPro), as opposed to its historical use as a rescue therapy after graft failure (secondary KPro). Specifically, for those patients with underlying conditions that have poor results with other forms of therapy including conventional corneal transplantation. In 2012, our center published the first documented case series on outcomes of primary KPro implantation[13]. Two other major artificial cornea centers followed suit with their own outcome studies, supplementing our data by conducting a short term comparison to secondary KPro implantation and focusing on the non-autoimmune disease population subset, respectively[14,15]. Compiled together, the primary KPro literature to date demonstrates rapid and sustained visual rehabilitation, adequate retention, and overall more favorable outcomes than penetrating keratoplasty (PK) for high risk patients[5,6,8,14–16].

Currently, there is a paucity of long-term data that directly compare the outcomes of primary and secondary KPro. Recent studies compared the pooled outcomes of primary and secondary KPro suggests that patients receiving primary KPro are more likely to achieve and maintain improvement in their visual acuity in comparison to patients undergoing a secondary KPro[14,15,17]. However, significant variability existed in terms of diversity of underlying diagnoses, follow up time, and the clinical setting in which the surgeries were performed. Patients in the secondary groups were not matched in baseline characteristics or diagnosis with the primary group. In general, potential candidates for primary KPro implantation tend to have more severe forms of ocular surface disease, e.g. Stevens-Johnson Syndrome, chemical burn, etc. In comparison, most patients with non-inflammatory conditions who are included within the best prognostic category would only be considered for KPro after many graft failures. As it is well established that perioperative diagnosis is one of the most important prognostic factors in KPro[8,9,14,15,18], a comparison of primary versus secondary KPro would be most useful when controlling for this variable[17]. The purpose of our study is to evaluate the outcomes of primary KPro with a matched group of patients who underwent secondary KPro.

## Methods

This research has been approved by the University of Illinois Office for the Protection of Research Subjects and has been conducted according to the principles expressed in the Declaration of Helsinki. All data were fully anonymized before access by the researchers and informed consent was waived by the Office for the Protection of Research Subjects. A retrospective, matched cohort study was conducted based on a chart review on all patients who underwent KPro (Boston type 1 keratoprosthesis, Woburn, MA) implantation at the University of Illinois Eye and Ear Infirmary (IEEI) by two artificial cornea surgeons (J. de la C., M.S.C.) between July 2008 and October 2014.

### Data collection and study design

This study included all eyes of patients older than 18 years of age with irreversible corneal visual loss who underwent KPro implantation. Patients were excluded if they did not meet the minimum follow-up requirement of 12 months. Primary KPro was defined as implantation of keratoprosthesis as the primary penetrating procedure in an eye with no prior corneal transplantation (patients who had prior cataract surgery, keratolimbal allograft transplantation and filtering procedures were still considered within the primary group). In contrast, secondary KPro was defined as implantation of keratoprosthesis in eyes with at least one prior corneal graft. Primary KPro was considered suitable for eyes that had severe corneal opacification which was unlikely to respond favorably to penetrating keratoplasty as the primary penetrating procedure due to one or more of the following criteria: extensive neovascularization (≥ 2 quadrants), advanced limbal stem cell deficiency (LSCD) (autoimmune disease, chemical/thermal burn, aniridic keratopathy), severe neurotrophic state (e.g. postherpetic anesthesia) and /or hypotony. Eyes with known end-stage glaucoma or end-stage vitreoretinal diseases were not considered appropriate candidates for KPro implantation. Data on preoperative characteristics (demographics, primary diagnosis, indication for surgery, comorbid conditions, baseline exam findings) as well as the intraoperative (device model and design, concomitant surgical procedures, complications) and postoperative course (exam outcomes, retention, complications, treatment) was extracted from the medical records. All eyes that underwent KPro implantation as the primary procedure that met our inclusion criteria were considered in the primary KPro group.

Following data collection, primary KPro eyes were then matched in a blind manner with eyes that underwent secondary KPro based on the following parameters in a progressive manner: 1) preoperative diagnosis 2) age 3) preoperative glaucoma and 4) KPro model (aphakic/pseudophakic) from a pool of 129 eyes. In the event that more than one eye from the secondary group was found to be a suitable match for a specific primary KPro eye, then the one closest in age was chosen to form the pair. Conversely, in the event that no eye within the secondary KPro pool was found to match based on a specific preoperative diagnosis then an eye with a similar diagnosis within the broader diagnostic category as described by the keratoprosthesis consensus group (e.g. recurrent immunologic rejection, chemical injury, and autoimmune disease) [19]. The investigator performing the matching process was blind to all patient data and outcomes except for the 4 parameters listed above.

### Examination and surgical procedure

All patients underwent a complete evaluation including detailed history; ophthalmological exam and B scan ultrasonography prior to surgery. The features of the Boston Type I KPro device, the standard surgical technique, and the postoperative management have been previously described in detail[1,20,21]. Preoperative glaucoma was determined by glaucoma specialists based evaluation of optic nerve, retinal nerve fiber layer OCT, and visual fields. Patients with concurrent glaucoma or retinal conditions underwent combined surgery consisting of pars plana vitrectomy, glaucoma drainage implant and Boston type 1 KPro as indicated.

### Outcome measures and data analysis

Best-corrected Snellen visual acuity and KPro retention were recorded as primary outcomes. Best-corrected Snellen visual acuity was then converted to logarithm of the minimum angle of resolution (logMAR) units. Count finger, hand motion vision were converted to 1.8 logMAR, 2.3 logMAR, respectively[17,22]. Patients were considered to have maintained their best-corrected visual acuity when their visions at last follow up was within 0.4 LogMAR units of their best-corrected post-operative visual acuity achieved. The data was then analyzed and reported using published consensus guidelines for reporting keratoprosthesis results[19].

Descriptive analysis was performed using Excel (Microsoft, Redmond, WA) and statistical analysis was performed using StatPlus (StatPlus, Version 6, AnalystSoft Inc., Walnut, CA). Chi-square test was used to analyze categorical data. Normality test was performed to determine if a data is normally distributed. Pre-operative and post-operative LogMAR visual acuities were determined to be normally distributed. Then, paired two-tailed t-test was used to compare visual acuities. Statistical significance was set at p < 0.05. Log-rank test was performed on Kaplan-Meier survival curves for maintenance of visual acuity and device retention.

## Results

### Patient demographics

A total of 56 eyes were included in this study. Twenty-eight eyes of 26 patients that underwent primary KPro implantation were matched with 28 eyes of 27 patients that underwent secondary KPro from a pool of 129 eyes. Of all eye pairs, 96% were successfully matched for preoperative diagnosis, 82% for age +/- 15, 82% for preoperative glaucoma, and 72% for aphakic or pseudophakic KPro model. We did not find a match for one patient in the autoimmune disease group based on diagnosis. However, this patient was matched with a similar patient (based on age, preoperative glaucoma, aphakic KPro model) with limbal stem cell deficiency due to thermal injury.

The average number of corneal transplants performed on eyes that underwent secondary KPro was 2.1 prior to undergoing a KPro. Table 1 lists baseline patient characteristics. The average follow up times were 5.0 years (range 1.1 to 8.6 years) for the primary group and 5.0 years (range 1.2 to 8.6 years) for the secondary group. Of note, 43% of the primary and secondary group had pre-existing retinal diseases that limited visual acuity recovery including foveal hypoplasia, retinal breaks, retinal detachments, epiretinal membranes, and vasoproliferative retinopathies. In addition, 46% of the primary group and 60% of the secondary group had pre-existing glaucoma.

**Table 1:** Baseline characteristics of eyes of patients who underwent Boston type I keratoprosthesis as a primary and secondary procedure.

Patient Characteristics	Primary KPro (n = Eyes)	Secondary KPro (n = Eyes)
**Follow Up (Years)**	5.0 ± 2.1	5.0 ± 2.9
**Mean Age (Years)**	49.3 ± 15.4	52.8 ± 16.4
**Number of Eyes**	28	28
**Number of Patients**	26	27
**Male/Female Gender**	50% (14) / 50% (14)	64% (18) / 36% (10)
**Caucasian Race**	61% (17)	67% (19)
**African American Race**	32% (9)	18% (5)
**Other Race**	7% (2)	14% (4)
**Preoperative retinal disease**	43% (12)	43% (12)
**Preoperative glaucoma**	46% (13)	61% (17)
**Average C/D ratio**	0.53 ± 0.15	0.52 ± 0.14

### Preoperative diagnoses

Preoperative diagnoses for all eyes are listed in Table 2. The majority of eyes (79%) required KPro implantation due to limbal stem cell deficiency of various etiologies including patients with severe chemical or thermal injuries, aniridia, autoimmune diseases such as Stevens-Johnson syndrome and graft-versus-host disease, and ectrodactyly–ectodermal dysplasia–cleft syndrome (EEC). In the chemical injury group 4 eyes (50%) of each group suffered from alkaline injuries; 2 eyes (25%) of each group suffered from acid injuries; 2 eyes (25%) of each group suffered from unknown chemical injuries. Four percent of eyes had neurotrophic corneas from severe herpetic eye disease. Only a minority of patients had hypotony (11%) due to uveitis and other reasons.

**Table 2:** Pre-operative diagnoses of eyes of patients who underwent Boston type I keratoprosthesis as a primary and secondary procedure.

Preoperative Diagnosis	Primary KPro	Secondary KPro
	n = Eyes (%)	n = Eyes (%)
**Chemical Injury**	8 (29%)	8 (29%)
**Aniridia**	7 (25%)	7 (25%)
**Autoimmune Disease**	5 (18%)	4 (14%)
**Gelatinous Corneal Dystrophy**	2 (7%)	2 (7%)
**LSCD/EEC**	2 (7%)	3 (11%)
**Infectious Keratitis/Neurotrophic Cornea**	1 (4%)	1 (4%)
**Hypotony/Uveitis**	3 (11%)	3 (11%)

### Surgical procedure

The majority of eyes underwent aphakic KPro (24 eyes in the primary group, 21 in the secondary group). Seven eyes in the primary and 9 eyes in the secondary group underwent concomitant glaucoma surgeries. In the primary group, these included 6 glaucoma drainage devices; in the secondary group, these included 7 glaucoma drainage devices and 1 shunt revision. Eight eyes of the primary group and 9 of the secondary group underwent concomitant vitreoretinal procedures. In the primary group, 6 eyes had pars plana vitrectomies; in the secondary group, 7 eyes had pars plana vitrectomies. Of the primary group, seven eyes had undergone cataract extraction prior to KPro implantation. Seven patients in the secondary group and one patient in the primary group had previously undergone keratolimbal allograft transplantation.

### Visual acuity outcomes

The preoperative mean best-corrected visual acuities were similar in both groups (LogMAR of 2.0 for both groups, p = 0.95) (Table 3). At postoperative years 1, 2, 3, 4 and 5 the primary KPro group attained better mean best-corrected visual acuity compared to the secondary KPro group. We observed a slow attrition of best-corrected visual acuity over the course of the follow up period with final mean LogMAR visual acuities of 1.2 for the primary group and 1.4 for the secondary group respectively (p = 0.21). Table 4 shows best-corrected Snellen visual acuities for both groups. A higher number of eyes in the primary group maintained 20/200 or better visual acuity at postoperative year 1 (71%), year 2 (67%), year 3 (64%), year 4 (67%) and year 5 (67%) in comparison to the secondary group (39% at year 1, 44% at year 2, 39% at year 3, 38% at year 4, and 29% at year 5. At the end of the study, 61% of eyes in the primary group and 39% of the secondary group maintained a final best-corrected visual acuity of 20/200 or better. It appears that at year 5, the primary KPro group had 1 NLP eye in comparison to none in the secondary KPro group. This difference could be attributed to the small number of eyes and is not statistically significant. However, it is also possible that the primary KPros were performed on worse cases and in patients with unknown retinal status preoperatively.

**Table 3:** LogMAR visual acuity outcomes.

	Preop Mean VA	Year 1 Mean VA	Year 2 Mean VA	Year 3 Mean VA	Year 4 Mean VA	Year 5 Mean VA
**Primary (n = Eyes)**	(28)	(28)	(27)	(22)	(15)	(15)
LogMAR VA	1.921 (±0.353)	0.833 (±0.368)	0.899 (±0.426)	0.830 (±0.591)	0.747 (±0.650)	0.899 (±0.625)
**Secondary (n = Eyes)**	(28)	(28)	(25)	(23)	(16)	(14)
LogMAR VA	1.927 (±0.324)	1.192 (±0.470)	1.263 (±0.640)	1.316 (±0.640)	1.558 (±0.795)	1.621 (±0.778)
**P-Value**	0.95	0.04	0.04	0.04	0.01	0.04

LogMar mean visual acuities for both primary and secondary groups measured pre-operatively, and at post-operative years 1 through 5. P-values were calculated using student T-test.

**Table 4:** Snellen visual acuity outcomes.

**Primary KPro**						
**Visual Acuity**	**Preoperative % (n = Eyes)**	**Postop Y1% (n = Eyes)**	**Postop Y2% (n = Eyes)**	**Postop Y3% (n = Eyes)**	**Postop Y4% (n = Eyes)**	**Postop Y5% (n = Eyes)**
≥ 20/50	0%	25% (7)	19% (5)	32% (7)	27% (4)	20% (3)
≥ 20/200	4% (1)	71% (20)	67% (18)	64% (14)	67% (10)	67% (10)
≥ CF	57% (16)	93% (26)	89% (24)	100% (22)	94% (14)	94% (14)
≥ LP	100% (28)	100% (28)	100% (27)	100% (22)	94% (14)	94% (14)
NLP	0%	0%	0%	0%	6% (1)	6% (1)
**Secondary KPro**						
Visual Acuity	Preoperative (#)	Postop Y1 (#)	Postop Y2 (%)	Postop Y3 (%)	Postop Y4 (%)	Postop Y5 (%)
≥ 20/50	0%	11% (3)	16% (4)	9% (2)	6% (1)	7% (1)
≥ 20/200	0%	39% (11)	44% (11)	39% (9)	38% (6)	29% (4)
≥ CF	54% (15)	89% (25)	84% (21)	83% (19)	63% (10)	50% (7)
≥ LP	100% (28)	100% (28)	100% (25)	100% (23)	100% (16)	100% (14)
NLP	0%	0%	0%	0%	0%	0%

Percent of eyes with each best-corrected Snellen visual acuities measured pre-operatively, and at post-operative years 1 through 5 in both primary and secondary groups.

**Fig 1:**
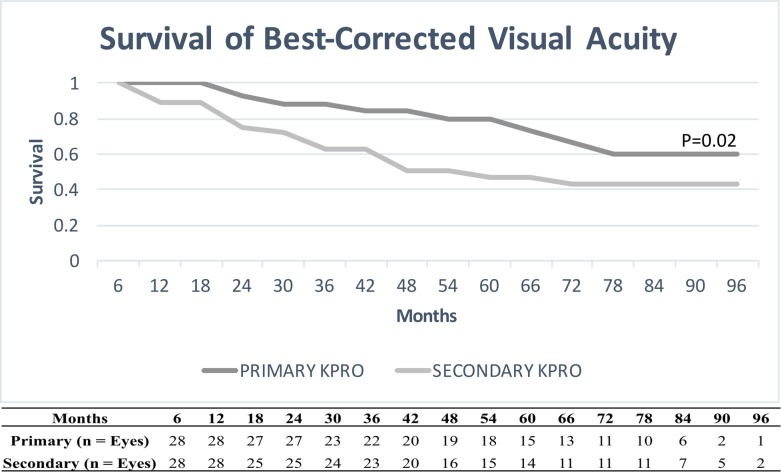
Survival of visual acuity. Graph showing best-corrected visual acuity survival curves for both primary and secondary groups. X-axis demonstrates post-operative months, and y-axis demonstrates survival of best-corrected visual acuity (e.g 1.0 = 100% of the eyes maintaining best-corrected post-operative visual acuity). P-value was calculated using Log-rank test. Table below shows the number of eyes followed at each time point.

Fig 1 illustrates the maintenance of best-corrected postoperative visual acuities in the primary and secondary groups as a survival plot. The probability for maintaining best-corrected postoperative visual acuities is 0.79 for the primary group and 0.49 for the secondary group at 5 years (p = 0.02).

### Device retention

**Fig 2:**
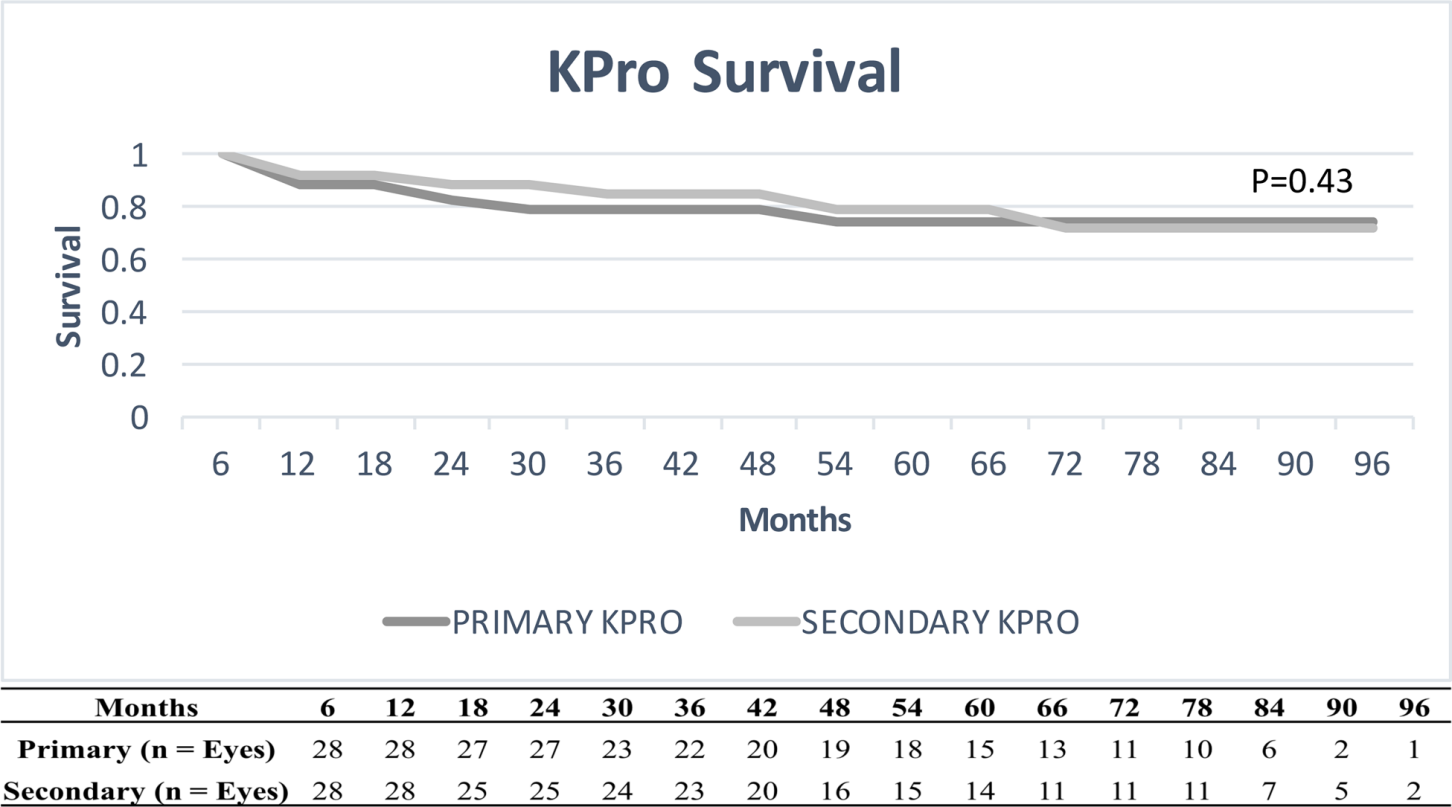
Device retention. Graph showing KPro survival curves for both primary and secondary groups. X-axis demonstrates post-operative months, and y-axis demonstrates KPro survival (e.g. 1.0 = 100% of the eyes with device retention). P-value was calculated using Log-rank test. Table below shows the number of eyes followed at each time point.

Fig 2 illustrates the survival analysis for device retention in primary and secondary KPro. There was no significant difference in the KPro failures rates (0.032 per eye-year for primary group and 0.034 per eye-year for secondary group, p = 0.43).

### Complications

Table 5 lists the postoperative complications developed in eyes implanted with primary and secondary KPro. It appears that postoperative complications, including sterile corneal melts, retroprosthetic membrane (RPM) formation, endophthalmitis, cystoid macular edema (CME), epiretinal membrane (ERM), rhegmatogenous retinal detachment (RRD) and hypotony were comparable in both groups. Of note, the percentage of eyes with new onset glaucoma (36% primary vs. 14% secondary, p = 0.06), worsening of existing glaucoma (32% primary vs. 25% secondary, p = 0.55), and needing post-operative glaucoma surgery (29% primary vs. 14% secondary, p = 0.19) appear to trend higher in the primary KPro group although no statistically significant difference was found.

**Table 5:** Complications.

Complications	Primary KPro	Secondary KPro	P-value
	% (n = Eyes)	% (n = Eyes)	
**Corneal Melt**	14% (4)	11% (3)	0.80
**RPM**	46% (13)	39% (11)	0.59
**New Onset Glaucoma**	36% (10)	14% (4)	0.06
**Worsening of Existing Glaucoma**	32% (9)	25% (7)	0.55
**Postoperative Glaucoma Surgery**	29% (8)	14% (4)	0.19
**Vitreoretinal disease progression**	21% (6)	14% (4)	0.49
**Endophthalmitis**	11% (3)	14% (4)	0.69
**CME**	21% (6)	14% (4)	0.49
**ERM**	21% (6)	11% (3)	0.28
**RRD**	11% (3)	11% (3)	1.00
**Vitreous Hemorrage**	4% (1)	0% (0)	0.31
**Vitritis**	4% (1)	7% (2)	0.55
**Hypotony**	11% (3)	11% (3)	1.00
**Choroidal Effusion**	7% (2)	11% (3)	0.64
**Choroidal Detachment**	4% (1)	0% (0)	0.31

Cumulative incidence of postoperative complications in eyes of patients who underwent KPro as primary or secondary procedure. P-values were calculated using chi-square test.

## Discussion

Boston type 1 keratoprosthesis is an increasingly popular option for patients with poor prognosis for traditional keratoplasty. When to offer this procedure in the course of corneal disease is still a matter of discussion as we lack evidence to support clear indication guidelines. However, there is a group of patients, particularly those with bilateral limbal stem cell deficiency and other severe forms of ocular surface disease that may benefit from this procedure early. Despite the need for good randomized clinical trials that compare treatment options in these patients, there is some evidence that suggests that in fact KPro may have better outcomes than other forms of surgical therapy[18,23–25]. Our study shows that KPro implantation as the primary procedure has good long-term retention and visual acuity results. Furthermore, it suggests that earlier intervention in a specific subset of patients may result in better visual outcomes when compared to KPro as a secondary or rescue procedure without an increased risk and/or severity of complications.

Eyes with KPro implantation as the primary procedure in our study achieved better visual acuity outcomes at postoperative years one through five in comparison to eyes that underwent KPro after prior corneal transplantation. Approximately 67% of eyes in the primary group maintained a best-corrected visual acuity of 20/200 or better at a follow up time of 5 years representing a substantial improvement from preoperative vision. In comparison, only 29% of eyes in the secondary group maintained a visual acuity of 20/200 or better at 5 years. It is important to keep in mind that a number of patients had pre-existing conditions that limited their visual recovery in this cohort. Visual acuity outcomes of this study are similar to previously published results from case series and non-case-matched studies. Large published case series report 56.5% to 100% of patients maintaining a vision of 20/200 or better after KPro implantation at various shorter follow up times than our study ranging from 8.5 months to 3 years[4,8,10,12,17,26]. Our case-matched study showed that 67% of the primary KPro group at 2 years and 67% at 5 years maintained 20/200 vision or better but only 44% in the secondary group maintained similar vision at 2 years. Most studies with longer follow-up observe a decrease in vision over time attributable to the development of postoperative complications [4,8,10,12,17,26]. Although the exact reason for the difference in visual acuity outcomes between primary and secondary KPro in our study is unknown, it is possible that complications that occurred after prior procedures may limit vision or predispose to progression of existing diseases such as glaucoma, though we did not specifically compare the severity of glaucoma for the two groups.

Our survival analysis suggests that retention of secondary KPro seems to be better than primary KPro at 2 years with higher number of eyes in the primary group requiring device explantation sooner. It is possible that patients who qualified for primary KPros suffered from more severe disease in comparison to patients who underwent a secondary KPro. This difference in severity of disease may have led to this initial difference in the rate of device retention. However, this gap narrows and at 3 years, KPro survival rates for both groups were similar with no difference found at the end of the follow up period between the groups. It is also notable that stem cell deficiency from various causes makes up the majority of the patients in our study (79% of eyes in both groups) representing the group of patients with highest risk of corneal melt and extrusion. Despite this concern, KPro implantation has the additional advantage of avoiding systemic immunosuppressive therapy required for allografting procedures in this group of patients. It was previously reported that the 5-year survival for allogenic limbal transplantation is less than 40%[27–29]. In contrast, recent studies looking at long-term outcomes of Boston KPro in the management of bilateral limbal stem cell deficiency have demonstrated promising results[18,23]. This study demonstrates a survival rate of 75% at 5 years for primary KPro. Thus, further validating the use of KPro as a reasonable option in patients with severe limbal stem cell deficiency related ocular surface disease.

It is well known that both KPro implantation and repeat PKs are associated with high rates of elevated IOP and glaucoma. In keratoprosthesis, the management of glaucoma is also challenging due to the difficulty in measuring IOP.[30] Previous studies have reported incidences of glaucoma requiring glaucoma surgeries after KPro implantation in the range of 15% to 35% at 5 years[17,31]. In this study, 25% of eyes in the primary group and 32% of eyes in the secondary group underwent concomitant glaucoma surgery at the time of KPro implantation. Although statistical significance was not reached, it appears that there is a trend for higher new onset glaucoma in the primary KPro group (36% vs. 14%, p = 0.06). The reason for this discrepancy is unclear. It is possible that this occurred by chance due to small number of eyes included in this study. It is also possible that in the secondary group, patients who had already developed advanced and end-stage glaucoma were excluded from the study, thus this may create a skewness in the homogeneity in glaucoma susceptibility in the secondary KPro group. However, it may be advisable for the surgeon to consider more liberal placement of concomitant glaucoma drainage device in eyes undergoing primary KPro implantation even in the absence of glaucoma.

The development of other major complications in the primary and secondary groups was comparable. Similar percentages of corneal melt, RPM formation, CME, ERM, RRD, vitritis, endophthalmitis and hypotony were found. The incidence of endophthalmitis in our study was 11% for the primary group and 14% for the secondary group. The high frequency of endophthalmitis may be attributed to the increased severity and complexity of ocular surface disease in the patients included in the study. This frequency of endophthalmitis was actually comparable to a previous large meta-analysis, which revealed a 5-year incidence of 10.3% for all patients undergoing KPro and 14.3% for patients with one prior graft[17].

This study has several limitations. First, the small sample size. In addition, the study is not powered to determine what specific preoperative patient profile or diagnoses will predict a successful outcome with a primary KPro implantation as oppose to another corneal procedure first and a secondary KPro later. Given the retrospective nature of the study, we were unable to fully match each eye in all parameters and develop more rigorous criteria. We were also unable to match primary disease’s severity and severity of comorbid conditions. In the future, a large randomized controlled trial may help to evaluate the outcomes of primary KPro, secondary KPro, and PKP +/- limbal stem cell transplantation.

## Conclusions

Our evidence supports the use of KPro implantation as the primary corneal procedure in patients with severe corneal and ocular surface disease. By matching patients with similar preoperative diagnosis and demographics, our study is able to more specifically compare outcomes between primary and secondary KPro. We showed that in a matched-case control setting, primary KPro patients achieved superior visual recovery and maintenance of post-operative visual acuity than secondary KPro.

## Supporting information

S1 File Primary KPro and secondary KPro patient profile. Table listing demographical and surgical characteristics of all patients included in the study.
(XLSX)
